# The impact of Hurricane Maria on individuals living with non-communicable disease in Puerto Rico: the experience of 10 communities

**DOI:** 10.1186/s12889-022-14552-4

**Published:** 2022-11-15

**Authors:** Elizabeth L. Andrade, Amalis Cordova, Courtney Riggle-van Schagen, Megan Jula, Carlos E. Rodriguez-Diaz, M. Ivonne Rivera, Carlos Santos-Burgoa

**Affiliations:** 1 Department of Prevention and Community Health, Milken Institute School of Public Health, George Washington University, 950 New Hampshire Ave, Washington, D.C, NW 20052 USA; 2 Rivera Group, 8206 17th Avenue, Hyattsville, MD 20783 USA; 3 Department of Global Health, Milken Institute School of Public Health, George Washington University, Washington, D.C, USA

**Keywords:** Natural hazards, Disasters, Impact, Hurricane, Non-communicable disease, Puerto Rico

## Abstract

**Background:**

Coinciding with the rising non-communicable disease (NCD) prevalence worldwide is the increasing frequency and severity of natural hazards. Protecting populations with NCDs against natural hazards is ever more pressing given their increased risk of morbidity and mortality in disaster contexts. This investigation examined Hurricane Maria’s impacts across ten lower SES municipalities in Puerto Rico with varying community characteristics and hurricane impacts to understand experiences of supporting individuals with NCD management in the six-month period following the hurricane.

**Methods:**

We conducted 40 qualitative interviews with mayors, first responders, faith leaders, community leaders, and municipal employees from 10 municipalities in Puerto Rico. Using QSR NVivo software, we deductively and inductively coded interview transcripts and undertook thematic analysis to characterize community-level hurricane impact and consequences for NCD management, and to identify convergent and divergent themes.

**Results:**

Damages to infrastructure, including healthcare facilities and roadways, complicated the provision of timely health care for NCDs, patient transport, and pharmaceutical/medical supply chain continuity. Lengthy power outages at both healthcare facilities and private residences were barriers to healthcare service delivery, use of medical equipment, and storage of prescription medications with refrigeration, and led to a widespread mental health crisis. Cascading failures such as fuel shortages further compounded these challenges. The consequences of these impacts included the reported exacerbation of health conditions and loss of life among NCD patients.

**Conclusions:**

Study findings identify contributors to morbidity and mortality among individuals with NCDs following Hurricane Maria. With the growing frequency of catastrophic disasters from natural hazards, the experiences of communities that endured these impacts offer important lessons regarding policies and practices to better support community disaster resilience and address the evolving preparedness needs of NCD patients.

## Background

In the aftermath of disasters from natural hazards, populations with non-communicable diseases (NCDs) are at disproportionate risk of morbidity and mortality, yet efforts to address the needs of individuals with NCDs in disasters have not kept stride with global climatological, demographic and epidemiological shifts in recent years [1–8]. This disparity has been evident following numerous disasters in recent U.S. history, including Hurricanes Charley, Frances, Ivan, and Jeanne in 2004, Katrina in 2005, Sandy in 2012, Irma, Maria, and Harvey in 2017, the west coast wildfires of 2017, 2018, and 2000, the COVID-19 pandemic beginning in 2020, and the Texas winter storm in 2021, to name a few [9–21]. This issue is also of particular relevance given that NCD prevalence and risk factors are on the rise in the U.S. and worldwide [22–24]. With a rapidly aging population [25–27], Puerto Rico has a relatively high prevalence of NCDs, including diabetes (16.7%), obesity (32.8%), asthma (18.1%), cardiovascular disease (7.2%), depression (17.9%), and chronic kidney disease (31.6%) [28, 29]. Coinciding with these trends, and compounding risk for individuals with NCDs, are the increasing frequency and severity of natural hazards [30–34] combined with the aging infrastructure of communities across the U.S. [35–41]. Given these trends, protecting populations with NCDs against natural hazards is ever more pressing [1, 2, 42–47].

Patients who suffer from NCDs often have more complex healthcare needs, and the capacity to adapt for adequate management of NCDs in disaster contexts is, in part, determined by socioeconomic status (SES) [48]. SES refers to the class or standing of an individual or group, and is frequently estimated using proxy measures of income, education and employment. Individuals of lower SES are more likely to be vulnerable to disaster impacts and face additional barriers to recovery than their higher SES counterparts. Ye and Aldrich (2019) found that SES was negatively associated with mortality following the 2011 earthquake and tsunami, asserting that lower SES residents were more likely to reside in lower quality structures, have less access to disaster-related information and preparedness trainings, and experience barriers to evacuation and response. Similarly, Kendig (2012) noted that precarious economic circumstances contributed to barriers in obtaining preparedness supplies, limited mobility, and residence in a high-risk area for disaster impacts.

Individuals with NCDs also rely on critical healthcare and community infrastructure including electricity, potable water, communication systems, and supply chains. In disaster contexts, populations with NCDs tend to be at greater risk of excess morbidity and mortality due to interruptions in these services and cascading failures in infrastructure that exacerbate their conditions by disrupting treatments, rendering medical devices inoperable, interrupting communication with healthcare providers, and limiting access to specialized diets, prescription medications and medical supplies [3, 18, 44, 46, 49–62]. Loss of electricity, for example, can result in downstream health impacts for patients with a number of different NCDs, creating challenges to obtaining dialysis, storing refrigerated medications like insulin, and operating life-support machinery like respirators, and filling prescription medications for mental health conditions [37].

As seen in Puerto Rico following Hurricane Maria, system failures are more likely following large-scale disasters and with aging infrastructure like electrical grids, placing communities across the U.S. at increased risk for these impacts [35, 36]. Individuals with NCDs who are victims of large-scale, catastrophic disasters that destroy critical infrastructure are at even greater health risk, in particular due to the lengthier time required for recovery of these systems [10, 12, 13, 46, 50, 63–65]. However, these more severe impacts and longer recovery time are not reflected in current preparedness guidelines for individuals with NCDs. Hurricane Maria, which devastated Puerto Rico by causing major damage to infrastructure, flooding, landslides, extended electrical and telecommunications outages, and disruption to health services delivery [52, 66–70], was also followed by a lengthy and problematic recovery [71–74]. While the risk of disaster-related death is acutely higher in the event’s immediate aftermath, patients with NCDs also have increased risk of death for a more prolonged period after a disaster has passed. Studies have shown that individuals with NCDs, including chronic kidney disease, diabetes, and mental health disorders, were severely affected following the 2017 Hurricane Maria in Puerto Rico [43, 75–81], with one study estimating that the leading causes of death after Hurricane Maria were due to complications from NCDs such as diabetes, cardiovascular disease, and Alzheimer’s [75]. Further, Acosta and Irizarry (2018) found that there was a prolonged increase in the death rate for circulatory, endocrine and respiratory causes of death - while the death rate increased by 74% immediately following the storm, a 22% increase was sustained for eight months post-hurricane. Similar to the findings by Acosta and Irizarry (2018), our research team from the [UNIVERSITY] estimated an overall excess mortality of 2975 from Hurricane Maria between September 2017 and February 2018 [82, 83], with older adults and residents of municipalities with lower SES experiencing disproportionate excess deaths and elevated death rates for longer during that time period [82, 84]. Based on these findings, this investigation sought to examine Hurricane Maria’s impacts in ten lower SES municipalities in Puerto Rico with varying characteristics to understand the nature of these impacts and community experiences of supporting individuals with NCD management in these post-hurricane contexts. As the frequency and severity of disasters from natural hazards increases, understanding the experiences of managing NCDs in the months following catastrophic disasters can provide important guidance to improve community and health system resilience and preparedness for future disasters.

## Methods

### Approach

For this investigation, we used a deductive approach to identify populations that were most vulnerable to the hurricane’s impacts and to understand how the health and well-being of these populations were affected in the context of these impacts. We used an inductive, grounded theory methodology [85] to conceptualize “disaster impact” based on the perspectives of diverse stakeholder participants from affected communities. The rationale underlying an examination of disaster impact is conceptually rooted in the potential influence that impact type [86], sequelae [87], magnitude, and duration of effects have on determining health outcomes following disasters.

### Participant selection and recruitment

In order to construct a participant selection frame, we created a municipal database that included categorical community attribute variables based on data sources found in Table 1 (the term ‘community’ will be used interchangeably with ‘municipality’). Attribute values were categorized to facilitate a selection process that ensured communities with a broad diversity of attribute values were ultimately selected. Categorization of attributes was also intended to support the interpretation of study findings by outlining the range of values that were typical in the context of Puerto Rico.

**Table 1:** Community attribute variables and data sources

Categorical Variables	Values	Data Source(s)
Region	Northern (N), Southern (S), Eastern (E), Western (W), Central (C)	Regional Map [88]
Terrain	Coastal, inland/mountainous	Topographic map [89]
Socioeconomic index (SEI)	Very Low SEI (39.0–47.5) Low SEI (47.5–50.6) Moderate SEI (50.6–55.1) High SEI (55.1–59.2) Very High SEI (59.2–82.5)	Índice de Desarrollo Socioeconómico Municipal [90]
Change in death rate post-hurricane (%)	Decrease (− 38.6–0) Very Minimal Increase (0–9.0) Minimal Increase (9.1–15.0) Moderate Increase (15.1–20.0) Severe Increase (20.1–28.3) Very Severe Increase (28.4–77.9)	Santos-Burgoa et al., 2018 [81]; Instituto de Estadísticas de Puerto Rico [91]; Meléndez & Hinojosa [92]; U.S. Census Bureau [93]
Change in pop. Density post-hurricane (%)	Very Minimal Decrease (− 2.0- -12.0) Minimal Decrease (− 12.1- -16.0) Moderate Decrease (− 16.1- -22.6) Severe Decrease (− 22.7- -36.0) Very Severe Decrease (− 36.1- -275.1)	Santos-Burgoa et al., 2018 [81]; Instituto de Estadísticas de Puerto Rico [91]; Meléndez & Hinojosa [92]; U.S. Census Bureau [93]
Hospital Access (min.) *(Calculated average travel time in minutes to nearest hospital)*	Very High Access (7.5–15.2) High Access (15.2–21.5) Moderate Access (21.6–28.7) Low Access (28.7–37.0) Very Low Access (37.1–61.1)	open street map [94]; DHS geocoded hospital dataset [95]; population density [96]
Dialysis Access (min.) *(Calculated average travel time in minutes to nearest dialysis facility)*	Very High Access (9.7–15.7) High Access (15.8–20.3) Moderate Access (20.4–27.0) Low Access (27.1–38.0) Very Low Access (38.1–77.0)	open street map [94]; CMS dialysis facility database [97]; population density [96]

**Fig. 1:**
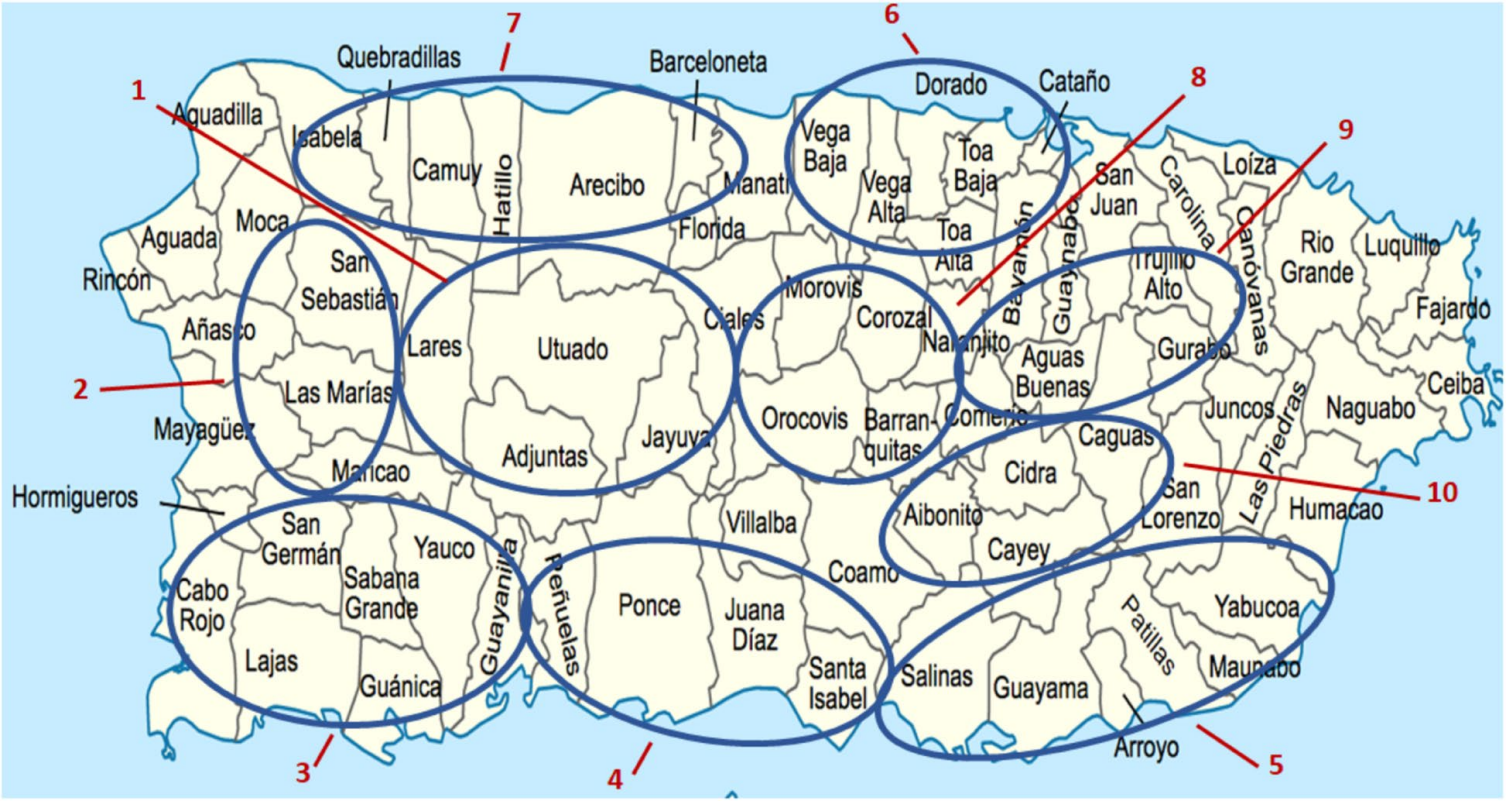
Map of selected communities

All municipalities included in the selection frame had low or very low SES [88], per findings from our prior research indicating that lower SES municipalities experienced disproportionate excess deaths following Hurricane Maria. Two variables pertained to community attributes that we anticipated would influence the type or magnitude of hurricane impact experienced (e.g., coastal vs. mountainous terrain, or geographic region), while two variables we anticipated would potentially influence a community’s ability to respond to health needs post-hurricane (i.e., pre-hurricane access to hospital or dialysis facilities). Two variables – change in death rate and change in population density from pre- to post-hurricane (a proxy for emigration) – were drawn from multiple data sources and our teams’ prior analyses. The selection of these variables was based on our prior research, which suggested that while all communities in Puerto Rico were affected by the hurricane, communities in different geographic areas experienced potentially differential impacts based on the trajectory and entry point of the hurricane, and that local disaster response was important since so many communities were cut off from outside aid due to the level of devastation – hence, variables of average travel time to hospital and dialysis facilities (proxies for service access) were included in the selection frame. While there are other potentially important factors that would likely determine NCD management, such as family support or having a regular source of care, data for these variables were not readily available at the municipal level. The intention of creating this selection frame was to enable purposive selection of 10 communities that were diverse, yet balanced, in terms of regional representation, terrain, and baseline healthcare access, and would potentially have had diverse post-hurricane experiences. In order to protect participant privacy, the exact selected municipalities are obscured, and instead, the general geographic location of communities are indicated in Fig. 1. Prior participation of the municipality in our initial 2018 research study was the only exclusion criteria [83].

We conducted in-depth, semi-structured qualitative interviews with 40 individuals across stakeholder groups, a sample size that was established in our prior research as sufficient to reach saturation regarding community-level experiences [83]. It should be noted that these stakeholder groups were sampled due to their broad knowledge and familiarity with the experiences and challenges of residents in their communities following the hurricane given their role managing/responding to the disaster or otherwise supporting residents. Thus, responses represent a second-hand account of the experiences of individuals with NCDs (referred to throughout as “experiences of individuals with NCDs”), as well as participants’ first-hand accounts of their efforts to support individuals with NCDs and the challenges they faced. Study participants were recruited by initially contacting the municipal mayor’s office to introduce the study and request an interview with the mayor. Additional participants were identified through referral by the mayor or other respondents. Interviews were scheduled by phone to take place in the local community in a private location.

### Data collection

We conducted interviews in 10 Puerto Rican municipalities with diverse characteristics. As seen in Table 2, selected communities varied widely in terms of their attributes pre-hurricane (baseline) and post-hurricane reported impacts (described more below). Approximately 4 interviews were conducted per community. Participants were recruited from the following stakeholder categories: municipal mayors (*n* = 9); first responders (*n* = 9); faith leaders (*n* = 5); community leaders (*n* = 9); and municipal employees (*n* = 8).

**Table 2:** Community pre-hurricane and impact attributes

Pre-Hurricane Community Attributes						Impact Attributes	
Community	Region	SES	Terrain	Hospital Access	Dialysis Access	Death Rate Increase	Pop. Density Decrease
1	C	Very low	inland	Very low	Moderate	Very minimal	Severe
2	C	Low	inland	Very low	Very low	Very severe	Very minimal
3	S	Very low	coastal	Moderate	Moderate	None	Moderate
4	S	Very low	coastal	Low	Low	Severe	Moderate
5	E	Very low	coastal	Low	Low	Minimal	Severe
6	N	Very low	coastal	Very high	Very high	Severe	Severe
7	N	Low	coastal	Moderate	Moderate	Very minimal	Minimal
8	C	Low	inland	Low	Very low	None	Moderate
9	C	Low	inland	Moderate	Moderate	Severe	Minimal
10	C	Low	inland	Very high	Very low	Severe	Severe

A semi-structured interview guide was developed to inquire about participant preparedness efforts as well as experiences during the response and recovery periods up to 6 months post-hurricane. Participants were asked about disaster communication and messaging, planning and decision-making processes, coordination efforts, and community engagement and collective actions pre- and post-hurricane. Participants were also asked to characterize the hurricane’s impact in their community, including consequences ranging from power outages and their duration [89] to impacts on resident morbidity and mortality. Additionally, participants were asked to identify the biggest threats to health and safety and the most vulnerable populations, as well as to self-rate their community’s resilience and identify strategies that most contributed to resilience (results related to resilience reported elsewhere). The interview guide was translated into Spanish adequate to Puerto Rico and pilot tested with one participant from our prior study. Interviews lasted approximately one hour each, and were conducted over a 17-day period in November of 2019 by a three-person bilingual research team with substantial qualitative research experience. Interviews were conducted in private locations in municipal buildings, community centers, or places of worship. All participants provided informed consent, and all research protocols were approved by the [UNIVERSITY] Institutional Review Board. All interviews were audio recorded and transcribed in Spanish.

### Data coding and analysis

Following a deductive approach, a codebook was created according to a priori areas of inquiry, including related to disaster preparedness and response effort coordination, the identification of vulnerable populations, and the biggest threats to health and safety. Spanish language transcripts were uploaded to QSR Nvivo version 12 qualitative data analysis software. The first 5 interviews were double-coded, with resultant coding compared to ensure concordance or identify needed code book modifications by combining, adding, or removing codes. The remaining 35 interview transcripts were coded by a primary coder and then all coding was reviewed by a secondary coder, after which any discrepancies were discussed and resolved.

During the coding process, using an inductive, grounded theory approach, we examined participant responses when asked to characterize the impact of the hurricane in their community. Based on these responses, we conceptualized “hurricane impact” domains, such as power, telecommunications water outages, which were described by participants as having an impact on resident health and well-being. Consequently, we created dichotomous variables at the community level based on participant responses regarding disaster impacts in these areas, to help with contextualizing and interpreting participant responses regarding consequences for populations with NCDs (See Table 3).

**Table 3:** Participant-reported community-level hurricane impact

Community	Electricity Outage	Water Outage	Telephone Outage	Internet Outage
1	> 6 months	> 3 months	> 6 months	> 6 months
2	> 6 months	> 3 months	> 6 months	Less than 6 mo.
3	Less than 6 mo.	> 3 months	Less than 6 mo.	> 6 months
4	Less than 6 mo.	> 3 months	> 6 months	> 6 months
5	> 6 months	Less than 3 mo.	Less than 6 mo.	> 6 months
6	> 6 months	Less than 3 mo.	> 6 months	> 6 months
7	> 6 months	> 3 months	Less than 6 mo.	Less than 6 mo.
8	> 6 months	Less than 3 mo.	Less than 6 mo.	> 6 months
9	Less than 6 mo.	Less than 3 mo.	Less than 6 mo.	Less than 6 mo.
10	> 6 months	Less than 3 mo.	Less than 6 mo.	n/r

Following the coding process, output by code was produced and reviewed to identify topics related to community-level impact and consequences for populations with NCDs, and to identify both common and divergent themes across interviews. We then thematically organized results by participant-reported impacts related to NCDs. Findings from the thematic analysis were summarized, and illustrative transcript segments were identified and translated to English.

## Results

### Hurricane Maria’s impact on NCD management and treatment

In the aftermath of Hurricane Maria, communities throughout Puerto Rico reported experiencing a number of challenges to supporting individuals in the management and treatment of NCDs. Many of these challenges stemmed from the magnitude of destruction of facilities, roadways, and public utilities, which created barriers to healthcare service delivery, resource access, and resulted in cascading failures, disruptions to supply chains, and a lengthy restoration of public services.

The results of this study will be presented in two overarching areas of hurricane impact: infrastructure damages and electrical power outages (See Table 4), and will illustrate a post-hurricane context in which NCD management and treatment was strained by numerous factors in those two areas: 1) Damages to infrastructure, including healthcare facilities and roadways, which complicated the provision of timely health care, patient transport, and pharmaceutical/medical supply chain continuity; and 2) Lengthy power outages at both healthcare facilities and private residences, which were barriers to healthcare service delivery, use of medical equipment, and storage of prescription medications using refrigeration, and led to a widespread mental health crises. Furthermore, results will also illustrate how cascading failures such as fuel shortages exacerbated these impacts across the board, resulting in additional challenges to the transport of patients to care or pharmaceutical/medical supplies to communities, and the powering of contingency electrical generators for sustained water supply, operation of medical equipment, and refrigerated storage of medications. Finally, the consequences of these impacts will also be discussed, in particular the loss of life that resulted from these circumstances. Direct participant responses are also included to illustrate findings.

**Table 4:** Overview of findings – hurricane impact and NCD management

Challenges:	INFRASTRUCTURE DAMAGES		ELECTRICAL POWER OUTAGES	
	Damaged Medical Facilities	Prescription Medication & Oxygen Supply Chain Interruptions	Storage Prescription Medications	Medical Facility & Residential
Adaptive Strategy:	• Travel greater distances for care • Move patients	Obtain Rx & oxygen in short supply	Restore power for the refrigeration of Rx	Contingency power generator
Barriers to Implementing Adaptive Strategies:	• Blocked roadways • Long travel distances • No telecom-coordinate care	• Blocked roadways • Damaged pharmacies	No contingency power generator	Dysfunctional or inadequate contingency generator
Cascading Failures:	Fuel shortages-transport patients	• Fuel shortages-transport supplies	• Fuel shortages- generator	• Fuel shortages- generator • Interrupted water supply from power outages • Lengthy power outages
Impacts:	• Delayed health care • Mortality from lack of services	• No Rx, oxygen obtained • Inadequate Rx, oxygen • Mortality-lack of Rx (cardiac, diabetes, mental illness), oxygen	• Unrefrigerated Rx became unusable	• Delayed health care • Inoperable medical equipment • Worsened mental health-power outages • Mortality-lack of medical equipment, dialysis, declining mental health, suicides

### The impact of infrastructure damages on NCD management

#### Damaged medical facilities

Infrastructure damages, including damages to medical facilities and roadways, were significant factors that led to delays in medical care for NCDs. Respondents identified types of facilities that sustained damage, compromising health care service delivery, including hospitals, clinics, medical offices, laboratories, dialysis treatment centers, and pharmacies. In two communities, damage to roofs of hospitals resulted in the decision to limit the scope of services and hours of operation:

*I witnessed the physical damages to the structure of the hospital. There were areas where the roof fell apart... we started to see how a part of the hospital was collapsing, all of that... we could operate the hospital, but operating it, I mean, in conditions that were not ok for it to be open. Even though the physical infrastructure wasn’t all ok, I had doctors, nurses, we could serve the population...other hospitals had collapsed entirely.*

-Mayor, Community #5.

*The disruption of services was unavoidable. The hospital suffered damages. At one point after the hurricane, we thought the hospital would have to be completely evacuated due to damage on the roof that flooded the emergency room, intensive care …*

-Emergency management personnel, Community #6.

#### Barriers to traveling to locate health services

With compromised health services, patients with NCDs needed to travel greater distances to locate operational services, but a number of communities described how this was impeded by inaccessible roadways due to debris, landslides, flooding, and collapsed bridges. Both individuals living in more remote areas as well as those who lived in town centers, but not on main thoroughfares, experienced lengthier times for roadways around their residence to be cleared. Two communities with low hospital access pre-hurricane perceived the inaccessibility of health services post-hurricane as one of the biggest threats to health and well-being.

While blockages from debris were reported in all communities, respondents in coastal municipalities reported flooding that took approximately 7–14 days to clear, with town centers being cleared earlier and outlying neighborhoods cleared later.

*As a matter of fact, [our municipality] was [in the top 10] most affected on the Island because some communities had flooding of more than 10 ft, those are communities that are very close to [the river] … - Mayor, Community #6.*

*Within a week and a half to 2 weeks, they were still clearing roads to reach the countryside. Within 7–8 days the urban sector was cleared. - Emergency management personnel, Community #3.*

Inland municipalities with mountainous terrain reported landslides that contributed to longer road blockages, sometimes lasting up to 3 weeks or longer.

*Well, in general all roads were obstructed. There were situations where more than a mile of [one] route had collapsed. You got there and you would say it was the end of the road, all the cliffs there, trees in the middle of the road...the first two weeks we were clearing small pathways. There was a [another] route that was closed for about a month.*

- Emergency management, Community #2.

*It was a long road and everything collapsed over that road. We spent 22 days with heavy machinery clearing that road. Around 2000 homes were affected, of which 700 were completely lost. I had a case of a large road that fell and that road went around and under these houses, a giant rock took with it homes with vehicles and animals inside, they fell into the river and disappeared. - Mayor, Community #1.*

*We had a neighborhood...we spent two weeks without being able to access that community. In another neighborhood, around two weeks as well to get to that community...but we had to do it with 4 × 4 vehicles, using alternate routes and back roads to get to those people and get them supplies. – Police officer, Community #8.*

Blocked roads were specifically identified as critical barriers to accessing healthcare for individuals with NCDs who needed regular services and treatments for management of their health conditions. Accessing dialysis treatment post-hurricane for patients with chronic kidney disease was of particular concern for study respondents, with seven communities mentioning dialysis treatment interruption as one of the biggest threats to health post-hurricane.

*Diabetics, those with heart conditions, those with high blood pressure. Those that could not go to hospital when they needed to due to factors we know, in terms of the blockages that there were … - Mayor, Community #6.*

*There were people that came here after 3–4 days because they could not receive dialysis, people that needed dialysis but because they were stuck, they came here.*

-Emergency management personnel, Community #2.

*The lack of transportation for dialysis. I had people here that arrived without dialysis treatment for 8 days … - Emergency management personnel, Community #4.*

#### Telecommunications outages

The barriers to accessing healthcare services from blocked roadways were compounded by limited communication to locate available services and coordinate care. Telecommunication service outages made it difficult for emergency managers, emergency medical personnel, and patients to communicate with each other to assess needs, coordinate care for individuals who lived remotely or had persistent roadway blockages from the storm, request medical transport, and call ahead to verify whether services were operational in neighboring communities.

*There was a situation with a patient where we had to find alternatives to reach them, to communicate because by that time, communications were a little better in terms of radio, not cellphones. That community was totally unreachable for communication …*

- Emergency management personnel, Community #1.

*There was no way of getting to the hospital, no way of going to Ponce because we didn’t, the vehicles, the sea, there were many things that we were examining and it wasn’t until we made the first trip and arrived in Ponce that we knew we could get there and verify whether they were going to receive patients or not. - Mayor, Community #3.*

#### Fuel shortages for medical transport

Fuel shortages were discussed as further complicating emergency medical transport of patients to receive services, and exacerbating NCDs.

*An Emergency Medicine supervisor arrived saying that the government did not have diesel for their ambulances. He asked me for diesel. As an agency, of course, yes, the ambulances are for the services of my people. But when I called them asking for services, they could not provide any... then that bad decision of not giving me those services was what made diseases worsen and people needed medical assessments.*

- Emergency management personnel, Community #4.

*Impacts: Delayed Health Care, Compromised Care Quality and Mortality.* Some study participants indicated that patients in need of health services did not seek medical care due to challenges they faced traveling long distances or the perception that facility conditions or quality of care would be compromised.

*Because it was difficult to go to the hospitals – since the one in our community was not operating, one had to go to [other cities], then it was harder for people. Many times … because they did not have transportation, they stayed, and did not get treated. Therefore, the situation worsened in terms of physicians here. - Community leader, Community #5.*

After roadways became passable, the discovery that medical facilities were closed led patients to travel to other communities in search of care, thus creating unpredictable shifts in service utilization patterns. One community discussed how patients had been diverted to their local dialysis center due to closures of other locations, and accommodating the increased patient load strained the center’s resources.

*In this region it was the only one, and patients were coming from other towns...so they started treating [the additional] people...to meet the demand, but this reduced the time each person could get dialysis. - Emergency management personnel, Community #1.*

Difficulties accessing healthcare services were cited as contributors to mortality among patients with NCDs, including cases where patients died in hospital parking lots or police stations due to severe respiratory distress, or died while in route searching for an operational medical facility or emergency medical care.

*I saw people die in the clinic parking lot... I had already closed the clinic. Family members took her because she could not breathe, but it was closed. They got an ambulance to see if they could take her somewhere else but, on the way, [her vital signs] crashed, [she] died... It’s something that I’ll have with me for the rest of my life, to see a person that arrives dead to the clinic, to see a friend, well not see her, but hear that she died the next day because there wasn’t a hospital open... these are things that you say: it is very painful to see that those things were happening. - Mayor, Community #5.*

*For me it was very painful that they died due to...as the days went on, they could not get the services they needed. – Faith leader, Community #6.*

*I do know people who were ill and their treatments were delayed, people who had to leave the country to continue their medical treatments... many people for whom the process was accelerated and they died earlier than they would have.*

- Mayor, Community #3.

Most communities that described challenges in accessing dialysis for patients in the aftermath of the hurricane also reported deaths of dialysis patients in their community due to treatment delays. Participants from municipalities with low or very low baseline dialysis access emphasized that they knew of many deaths among dialysis patients due to not receiving dialysis.

*As consequences of [the hurricane] because there are people who were getting dialysis and had to travel to other towns, there was no transportation, none. The area was not prepared to transport them and well, with the lack of medication and care, they died.*

- Firefighter, Community #2.

*It was mainly dialysis. I had to escort people that needed dialysis or had delayed their dialysis. We had to escort them to the nearest hospital, transporting people who were already the color lilac. They’ve now died... they didn’t pass away right after the hurricane - because their treatment was affected, eventually they died. Dialysis patients, I would say that here, totally related to Maria, over 100 cases. They were pretty high.*

- Mayor, Community #8.

#### Prescription medication and oxygen supply chain interruptions

Respondents from all communities highlighted interruptions to prescription medications and oxygen supplies as contributors to inadequate management of NCDs following Hurricane Maria, especially for diabetes, hypertension, cardiovascular disease, mental health disorders, and asthma. Furthermore, respondents from 4 communities perceived prescription medication shortages as one of the biggest post-hurricane health threats.

*Well, medications were not available, particularly for people with diabetes, insulin, for high blood pressure … - Mayor, Community #6.*

*Then, a person’s medication supply runs out, a hypertensive person, a cardiac patient, a diabetic person … for me, yes, the stress was a lot for a person that needed medication... and didn’t have it accessible. Here, thank God, the hospital was able to operate without stopping. But whoever needed access to medications, there were difficulties here. - Municipal employee, Community #10.*

*They ran out of medication... Then, well, she needed it, because she was older, and she needed that medication … honestly, it was an odyssey, because of what happened that week after Hurricane Maria... medications were what people asked for the most.*

- Police officer, Community #8.

In addition to interruptions in the supply chain due to road blockages and difficulty transporting medications to local communities, damages to pharmacies or closures were also identified as contributors to medication shortages. Participants from six communities reported that pharmacy services had to be temporarily suspended or offices closed entirely due to the damage.

*The pharmacy was non-operational for many weeks. - Municipal employee, Community #10.*

*We lost pharmacies … the impact was so great for our people. - Mayor, Community #3.*

Furthermore, study participants from six communities reported shortages in oxygen supply, which put patients’ lives in danger.

*I remember we had to, at one point, run to get oxygen tanks, somehow find people that had oxygen tanks they were not using because...or people whose saturation started to drop in reality had their lives in danger and at risk of death, and we had to do a lot of arrangements, lots of things to get those things because the state was not providing them. - Mayor, Community #4.*

*… I still get emotional remembering those moments (voice breaks) because of seeing people who needed oxygen … -Municipal employee, Community #10.*

*Impacts: Morbidity and Mortality from Interruptions to Medication and Oxygen Supplies.* Two respondents mentioned cases in their communities where lapses in access to medications for the management of mental health disorders contributed to declining wellness and even disappearance of patients.

*Many people didn’t have medications...for their mental health, critical. As a result of the lapse in medication for mental health, many people started to get depressed and arrived here crying. - Emergency management personnel, Community #4.*

*We had some particular situations that left a mark on us. We have a colleague that disappeared after Hurricane Maria, we still haven’t found her, a co-worker that was undergoing psychological treatment, and what happened? The doctor closes the offices after the hurricane and she ran out of medications and she disappeared. We don’t know where she is … search teams were made. We haven’t found her. - Municipal employee, Community #5.*

Respondents from six communities indicated that people had died in their communities due to shortages in medications to treat NCDs.

*Access to medications, that was fatal. In fact, I know people that shared their medications. - Faith leader, Community #7.*

*People didn’t die in the hurricane, they died after the hurricane, for many reasons: lack of electricity, oxygen, medications. - Community leader, Community #9.*

*And seeing whatever number of deaths, you realize that the majority of people died because of lack of medications. Couldn’t get their dose, sugar levels went up, and boom! - Emergency management personnel, Community #6.*

Furthermore, all communities reporting oxygen tank shortages also indicated that deaths had occurred in their community because of these shortages.

*In fact, people who were at the small hospital... many people died there as well because of a lack of oxygen, because sometimes if there weren’t generators, they couldn’t …*

- Municipal employee, Community #8.

*We had a case of a person, the family had their grandmother at the house, she did not have oxygen at the house but she could walk. The bad thing was that she had asthma. She had a stabilizing treatment. Then, the hospital was closed. Well, they took her to the office, to the municipal police, they sat her down at the police station and when our paramedic went to assess her, she was dead, sitting right there in our reception area, in front of us. - Mayor, Community #8.*

### The impact of electrical power outages on NCD management

#### Refrigerated storage of prescription medications

Further complicating the issue of prescription medications, respondents from seven communities identified power outages as a barrier to storing refrigerated medications, especially insulin for diabetes management.

*You have people that have some treatment that’s ongoing, that are diabetic, hypertensive... They don’t have electricity, don’t have a way to keep those medications refrigerated. - Mayor, Community #5.*

Among these communities, 5 reported power outages lasting longer than 6 months, and while the other 2 communities regained electricity in the urban center relatively quickly, they still reported extended outages in rural areas, with some rural neighborhoods not regaining electricity until up to a year post-hurricane.

*People with diabetes, their insulin would be damaged since there was no electricity. It was very difficult to go and give them a bag of ice because, where could you get ice? Where there was electricity. And where was there electricity? Well, in the town they say there were generators, but in the country side it was not common to see a functioning generator... everyone prepared, yes - I have insulin for this much time...but no one knew how much time the need and suffering would go on. - Firefighter, Community #2.*

#### Fuel shortages for medication refrigeration

These seven communities also described how fuel shortages created additional challenges for refrigerating medications, such as insulin, due to the need for diesel fuel to power generators.

*People who were bedbound and using refrigeration for medicine … there wasn’t. Then there was no gasoline, you had to go look over there and the lines were very long and you would get there in the morning and still there was a line at night and when you reached the station, they would say “there’s no more gasoline.”*

- Community leader, Community #1.

*Impacts: Unusable or Discarded Prescription Medications.* Given the lengthy power outages in many communities, participants reported that patients began to discard medications that could no longer be refrigerated, further limiting the already dwindling supply.

*Since they didn’t have electricity in their homes, when the insulin warmed up a lot … people began to throw it away … - Mayor, Community #3.*

#### Power outages in medical facilities and patient residences

Widespread power outages were described as a major barrier to healthcare service delivery, the use of medical equipment in patient homes, and contributed to water supply interruptions with implications for dialysis service delivery. To provide context to the extent of power outages among communities sampled for this study, seven communities reported being without electricity for longer than 6 months. Six of these communities mentioned the lack of electricity as one of the biggest post-hurricane threats to health and safety.

*Well, for me the biggest threat was access to medical services. Doctors’ offices were closed, pharmacies were closed, because there was no way to operate them, there was no electricity … - Municipal employee, Community #10*

...*they were sick and where were they going to go? Then, the clinics were the closest thing, but there was no electricity. There was nothing. We were not prepared.*

- *Faith leader, Community #7*

Study participants identified individuals who relied on the use of medical equipment in their homes as a population that was particularly vulnerable post-hurricane following power outages. Eight communities indicated that the extended power outages contributed to inoperability of ventilators or other medical equipment for patients with NCDs and complex medical conditions. Four communities, all with power outages lasting 6 months or longer, identified the inability to power medical equipment as one of the biggest threats to health and safety.

*Many people had some type of impediment, some kind of need... They would need some equipment, yes. I heard of many situations, where people did not have electricity, the generator wasn’t working. I believe there were many people who lost their lives in those areas. Many people were in their homes, and they needed special equipment, and the equipment required electricity. Then, when there was no electricity, they turned to generators, and if the generators failed at any moment for X or Y reason, then …*

- Municipal employee, Community #10.

*Like I said the situation around health, the majority of people that suffered here were those that needed some equipment, because of the electricity situation, people that were bedridden, that needed oxygen … many people passed away here. Like I said, the majority of people that died had preexisting conditions that were aggravated by María. Directly the day of impact, no, but as a consequence of the situation the hurricane left behind, many people worsened and consequently died. - Emergency management personnel, Community #5.*

#### Unreliable electrical power contingencies

Compounding the challenges related to power outages, three communities identified barriers to providing patient care due to problems with backup electrical generators for medical facilities. These problems ranged from generator failures immediately post-hurricane to sporadic/periodic health facility closures due to generator failures. In the case of one community, restoration of a failed hospital generator did not occur until almost one-week post-hurricane:

*There was no communication with the hospital administration until five days later. They notified us that the generator failed and there was no way to operate... The hospital was open the night of the hurricane, but after the hurricane passed, the hospital was closed. When I reached the hospital, they told me the generator failed. They had to close the hospital and [the municipality] was without a hospital for 6 days and everyone had to go to [another town] to tend to any emergencies. It was very tough. - Mayor, Community #9.*

Another municipality described the inability to power the local clinic for 24-hour service delivery due to the limited capacity of an old generator:

*In the clinic, we had a generator, which was an old generator, that we gave maintenance to be able to keep it operational … we were operating with a limited schedule. We couldn’t have the generator 24 hours because it was going to break on me. And I asked the people from FEMA to please find me another generator to be able to have the clinic working 24 hours, and it arrived 84 days after the hurricane. - Mayor, Community #5.*

One community described how the electrical generator for a refuge was inoperable, introducing a major barrier to individuals who needed electrical supply to operate life-sustaining medical equipment (Community 6).

*...factors related to electricity. On many occasions, considering that many people should have ventilators and faced with the fact that the shelters where they were located did not have a functioning generator, then, that limited on many occasions that service. - Mayor, Community #6.*

#### Fuel shortages for contingency power generators

Respondents from all 10 communities described diesel fuel shortages that began a couple weeks post-hurricane, which introduced another impediment to patient care since electrical generators that sustained the operation of water pumps and powered hospitals, clinics, and pharmacies required diesel fuel. Respondents described the additional complexity of balancing competing priorities for disaster response activities with limited supply, such as fueling emergency medical transport vehicles, vehicles for supply delivery, or to power generators. Consequently, diesel shortages, whether temporary or persistent, had an impact on the operational status of medical facilities, especially in more remote, inland municipalities.

*The first two weeks, the two clinics we had in the area were in operation, but once they ran out of gasoline... they closed, we had to go to another town … some 35 minutes to go to that secondary hospital facility where they were accepting [patients].*

- Emergency management personnel, Community #1.

*Many times, in the hospital they had to provide them with diesel for generators so they could keep working. It was not easy. It was very difficult 3 or 4 months after the hurricane. – Mayor, Community #1.*

Study participants described delays ranging from one week to one month to receive diesel fuel in their communities due to blocked roadways.

*Here, the biggest problems were regarding gasoline. Some families had generators, but gasoline was difficult, because roads were obstructed for those first trucks, they got here after a week. That first week, fuel was critical. -Municipal employee, Community #8.*

*For us, for fuel to be available in this area, we started seeing it around 10 days after because the road was split in half, we had to clean it so trucks could go up there.*

- Emergency management personnel, Community #2.

Additionally, shortages of fuel were reported by all 10 communities as creating challenges for powering medical equipment in patient homes. According to participants, few gas stations were receiving supply, lines were long, and strict rationing made it hard to obtain gasoline.

*There was a lady with hypertension, her husband went out to look for gasoline for their generator, but the lines to look for a little container of gasoline took 3 hours. That man was in line to keep his generator on, and when he arrived at home after 2 hours in line, he found his wife on the floor because she had collapsed. By the time he brought her to the hospital, she was already dead. Many cases like that. - Mayor, Community #5.*

#### Water outages from power failures compromised Dialysis treatment

Respondents from three communities mentioned the lack of electricity as a barrier to water supply delivery, indirectly affecting healthcare service delivery. A total of 5 communities reported being without tap water for more than 3 months, with variability between urban and rural areas.

*There were sectors that always had water, it would be cut off per hour when the water pump ran out of diesel but there were sectors that went 2 to 3 weeks without water... the more isolated sectors. - Emergency management personnel, Community #3.*

*Anything closer to the urban center, by November [2017] there was electricity and water... It was maintained in the town, here in the center, but for other sectors that depended on electricity for water pump systems … well, basically until February [2018].*

- Faith leader, Community #6.

*My last community got electricity within 7 months and the first by December [2017].*

- Mayor, Community #7.

One community described shortages in the bottled water supply, which required rationing of this resource.

*The little water that arrived, I remember like it was today, the mayor said we had to share because there were more than 50 thousand inhabitants, we have to share this water. And we went to houses with small bags and some water bottles to give a little to everyone. And people did not understand. They thought we were hiding them. And no, there was so little water that we could only give out 12 water bottles to give some to everyone. And that broke our hearts. - Community leader, Community #6.*

Another municipality described the challenges that water supply interruptions created for the nearby dialysis center, which needs a water supply to operate.

*We also had a situation here: the dialysis center had a processing plant, a plant where... a water treatment plant, but they ran out of water, we had to bring water to them.*

- Emergency management personnel, Community #1.

*Impacts: Mortality from Lack of Health Services, Mental Health Crises, and Inoperable Medical Equipment.* The recovery following Hurricane Maria was slow and extended, characterized by delays in restoring electrical power to communities. Long periods of time without power, combined with damaged homes and infrastructure, created a difficult living environment for many in Puerto Rico. Eight communities identified the decline in resident mental health as one of the biggest post-hurricane threats to health and safety, with respondents specifically mentioning concerns about depression, anxiety, and post-traumatic stress disorder.

*The panic, the panic, the nerves, many people sick with nerves needing to go to the hospital and be hospitalized. - Mayor, Community #7.*

*Older people, especially, are the ones that get depressed. A lot of depression. - Municipal employee, Community #8.*

*We had a lot of cases of depression, well, caused by trauma, right? Many people did not expect the impact we experienced. - Community leader, Community #5.*

Participant responses indicated that some individuals had pre-existing mental health disorders that were exacerbated post-hurricane, while other individuals experienced mental health crises as a result of the hurricane’s severe consequences and the lengthy recovery period that followed. Furthermore, study participants characterized the increases in mental health crises as being widespread, but particularly concerning among residents of communities with lengthy power outages. Three communities described cases where the hurricane’s impact, especially multiple simultaneous losses and extended electrical outages, contributed to residents’ decisions to commit suicide.

*Another thing in terms of health was the anxiety that it produced in the town. Suicide attempts, suicide that happened because of living in those conditions for so long and it was one of the situations that also greatly concerned us. In one day, two people committed suicide, on one occasion, one morning, they found a person who committed suicide in the morning and that night electricity came back to where he lived. - Mayor, Community #5.*

*On one occasion, we received information from social workers that worked in schools and they informed us that they were getting, all of the sudden, a lot of cases of kids that were expressing suicidal thoughts - with the desperation, without internet, without electricity, without food, it’s so hard. - Municipal personnel, Community #5.*

*The cases of suicide increased after Hurricane Maria. The people that could not deal with the situation … across all of Puerto Rico because it was not just here, all of Puerto Rico.*

- Emergency management personnel, Community #1.

*For me, what did take off were the suicides because in this position one finds out, one finds out every time there is a situation like this one and it got to a point where one says “My God, another one!” like... This is a small town, so when everyone knows each other and someone says, “hey, so-and-so took his life,” it shocks you. I believe it is related to the losses...many losses at the same time. - Municipal employee, Community #10.*

Eight communities reported that difficulty operating ventilators or medical equipment due to a lack of electricity or fuel for generators contributed to deaths in their community.

*Many deaths happened not as a direct cause of the rain, winds, rather with the passage of time. There were many people, many families lost fathers, mothers, people that were bedbound. And how are you going to say that that wasn’t... that it was only because of natural or medical circumstances, or that they were already sick? No, because a person that needs an oxygen machine in their home and they didn’t have electricity, they were going to die because they needed the oxygen. – Faith Leader, Community #3.*

*People who had respiratory conditions because they had to be connected to an oxygen system. I remember a young man that always needed that system. When the power went out, they looked for help to go to the hospitals, but all of the roadways were blocked because it was in the middle of the storm and he passed away.*

- Mayor, Community #9.

These deaths that resulted from the inability to operate medical equipment were mostly described as deaths that occurred in residents’ homes.

*Another situation we saw when we visited different communities with bedridden people that needed equipment or ventilators, electricity. When electricity collapsed for these people, many of those people died in their homes … but it’s not documented as a consequence of the lack of electricity and necessary equipment. - Municipal employee, Community #5.*

*Families had corpses in their homes for three days with ice, with the little ice they could find. The person did not receive any service. Electricity, needed equipment, oxygen - he didn’t have those. So, he died there. But we had to go get him, and it was a difficult process … We had nine, ten people that depended on equipment that eventually died.*

- Mayor, Community #8.

## Discussion

This study sought to understand how communities in Puerto Rico experienced the impacts of Hurricane Maria, and how these impacts and cascading failures in infrastructure and key resource sectors may have affected the management of NCDs. The communities selected for this study represented a great degree of heterogeneity in terms of underlying context and post-hurricane circumstances, helping to better understand the breadth of impacts, in particular among lower SES communities, which experienced disproportionate excess mortality following the hurricane. There were a number of populations that were characterized by study participants as being at highest risk for excess morbidity and mortality post-disaster, and they were identified by participants based on their: a) specific health conditions, including individuals with chronic kidney disease, diabetes, cardiovascular disease, respiratory disease, and mental health disorders; b) requirements for NCD management/treatment, such as continual or regular use of electricity-dependent medical equipment, oxygen, prescription medications, and/or other treatments; and additional factors that compounded risk, such as c) advanced age; and d) limited mobility. The vulnerability of these high-risk groups was influenced by damages to healthcare and community infrastructure, especially from high winds and flooding, and lengthy interruptions to public services, in particular electricity. Both of these types of impacts presented challenges to adequate management of NCDs, with persistent impacts of road blockages and power outages being major impediments to patient transport, continuity of medical supplies, and operation of life-saving medical equipment.

A majority of study communities identified barriers to dialysis as one of the biggest threats to health post-hurricane, either due to dialysis unavailability from center closures or center inaccessibility from impassable roadways. These findings are consistent with other studies that have documented the vulnerability of dialysis patients following disasters due to missed dialysis sessions, including as a result of transportation-related barriers; however, this study identified considerable variation in the length of time that roadways were blocked based on types of impacts that varied by terrain, highlighting the importance of considering these factors during preparedness for natural hazards of this magnitude.

Furthermore, guidelines for dialysis patients on how to prepare for natural hazards vary widely. Most publicly available guidelines would under-prepare patients in contexts of catastrophic disasters similar to those seen following Hurricane Maria. Current guidance would fall short of protecting patients experiencing severe damage to infrastructure and extended power outages. For example, some guidance relies on infrastructure and resources that are less likely to be available following severe events, such as reliance on telecommunications to coordinate care and verify service availability or the designation of primary and secondary dialysis centers without consideration of actual post-disaster roadway access conditions or other impacts. Guidelines for dialysis patients should be expanded to include information on how to prepare for scenarios following catastrophic disasters, including expanded options for local dialysis treatment that addresses transportation-related, roadway access barriers, and damage to dialysis centers that are likely to occur, and persist, post-disaster.

The vulnerability of the high-risk groups identified by study participants was also related to *key resource interruptions* post-hurricane, namely electricity, generators, diesel fuel, and potable water. Based on study participant responses, the resource interruption with, by far, the most pervasive negative consequences for NCD management and treatment was electricity, compounded by inadequate contingencies and cascading failures related to fuel supply. The consequences from power outages were felt across all study communities and were described as important factors that contributed to substantial morbidity and mortality among those with NCDs. Importantly, power supply and access to generators was identified as being vital both in clinical and home-based settings. A reliable electrical source was not only essential for the operation of medical and dialysis facilities and pharmacies, but also for the use of life-saving medical equipment and ventilators, often in patients’ homes. Individuals requiring dialysis and medical equipment were reported as being the most vulnerable, as most study municipalities reported deaths among these populations post-hurricane. This finding related to the impact of power outages on individuals with NCDs is consistent with other studies [44, 92–97]. However, this study found that the negative effects from electrical outages following Hurricane Maria extended far beyond the immediate post-hurricane period, with interruptions lasting, in many communities, months at a time. As reliance on generators extended over such a long period of time, so did the reliance on a stable supply of diesel fuel. As fuel shortages became increasingly pervasive, the operation of generators to power medical facilities and life-saving medical equipment was further complicated. Lengthy power outages were also reported by participants as severely affecting mental health and presenting significant long-term challenges for the refrigeration of medicines like insulin. While the effects of power outages on individuals with NCDs has been described in other studies, the extent and duration of these interruptions following Hurricane Maria far surpassed those previously reported [46, 78, 96, 98, 99], and the implications of *long-term* power outages for individuals with NCDs, including considerations of fuel supply sufficiency to power generators and mental health consequences, have not been adequately described in the literature. This finding is particularly relevant to individuals with NCDs living in communities throughout the U.S. with aging infrastructure and electrical grids.

Existing guidelines for patients with NCDs who are reliant on electricity do discuss how to prepare for power outages, but many consist of checklists or planning tools and don’t give specific recommendations for how to handle the indirect impacts from disaster, much less in severe or catastrophic disaster scenarios [100]. Additionally, some guidelines expect patients to have generators, but low-income populations experience greater barriers to purchasing generators and procuring a fuel supply for an extended period [1, 96, 101]. Overall, guidelines don’t recommend that patients prepare for a time period that is in line with the duration of impacts from catastrophic disasters, such as those seen following Maria, and tend to rely on resources that may not be available such as telecommunications, fuel to power generators, or ice to cool medications [102]. In the case of Hurricane Maria in Puerto Rico, communities had prepared, but not for the possibilities of experiencing power and telecommunication outages that persisted for months at a time, combined with shortages of diesel fuel and few alternatives to refrigerate medications.

Other key resource disruptions that were described as contributors to morbidity and mortality post-hurricane were related to interruptions in the *prescription medication and oxygen supplies*. All communities reported impacts from medication shortages, with individuals managing diabetes, mental health disorders, and hypertension reported as being most affected. Furthermore, six communities reported oxygen shortages and deaths related to this lapse in supply. While there are studies that have reported the impact of medication shortages on individuals with NCDs [20, 43, 44, 46, 79, 96–98, 103, 104], there are fewer studies that have reported similar findings related to oxygen supplies. Many current patient guidelines recommend storing a 3–7-day supply of medications and inform patients about prescription assistance programs, which require access to internet or telephone [4, 5]. However, given the experiences following Hurricane Maria, guidelines should be expanded to recommend that patients anticipate the possibility of longer service, transportation, and resource interruptions, and prepare accordingly for the possibility of catastrophic events, including how to handle medication or oxygen shortages for preparedness at the household level.

### Limitations

There are some limitations to this study that should be considered when interpreting results. First, 10 municipalities were selected out of 78 total municipalities in Puerto Rico, and one inclusion criteria was that the municipality had lower SES. The smaller number of lower SES communities limits the generalizability of findings to the broader population in Puerto Rico. In order to minimize this limitation, we used a sampling strategy to identify municipalities with a diversity of characteristics to increase the likelihood that participants would have a wide range of experiences in terms of hurricane impact. Importantly, low SES municipalities were the most severely affected following Hurricane Maria in terms of excess mortality [82], and study findings are likely applicable to other low SES communities and individuals given that 44% of Puerto Ricans lived in poverty as of 2019 [96, 105]. While this study does not facilitate an assessment of causal relationships between disaster impact and mortality, findings do provide insight into the factors surrounding morbidity and mortality of individuals with NCDs, as well as contributors to health care system and public service impacts that negatively affected NCD management.

## Conclusions

The experiences following Hurricane Maria in Puerto Rico offer important lessons regarding the potential impacts of catastrophic disasters on NCD treatment and management, and are useful for improving community and health system resilience and preparedness for future disasters. There were a number of populations that were identified by study participants as being most vulnerable to morbidity and mortality post-hurricane, in particular individuals with NCDs that required lifesaving medical equipment, dialysis, and prescription medications. These high-risk groups should be prioritized for future disaster preparedness planning, especially when multiple risk factors are present and there are additional financial barriers to adequate preparedness. The vulnerability of individuals with NCDs was influenced by the type and duration of cascading hurricane impacts. Catastrophic disasters introduce additional preparedness considerations for individuals with NCDs. Preparedness guidelines for individuals with NCDs should be expanded to consider these possible impacts as a result of catastrophic disasters, especially with the demonstrated increase both in the intensity of natural hazards and the proportion of the U.S. population with NCDs. When disaster impacts could involve considerable infrastructure damage that would limit travel to healthcare services or prevent communication with providers, preparedness plans and contingencies should anticipate the need for in-place primary and NCD care, including in local community and home-based settings. Community disaster preparedness plans should integrate local medical systems that support home-based care to enhance the timeliness and effectiveness of care for NCD patients. Furthermore, compromised treatment and management of NCDs due to the extended duration of interruptions to services and resource availability highlight the need for preparedness approaches that anticipate these longer periods. Preparedness guidance for pharmacies, medical offices, and specialized care units such as dialysis centers should consider planning for a network collaboration between different units and standards for equipment. Disaster preparedness guidelines for individuals with NCDs should be expanded to apply lessons learned from Hurricane Maria in order to protect populations with NCDs from excess morbidity and mortality in future disasters.

## Data Availability

The datasets generated and analyzed in this current study are not publicly available to protect participant privacy, but are available from the corresponding author on reasonable request.

## Abbreviations

GW: George Washington University Milken Institute School of Public Health
NCD: Non-communicable disease
SES: Socioeconomic status
